# TIGAR Protects Cochlear Hair Cells against Teicoplanin-Induced Damage

**DOI:** 10.1007/s12035-023-03309-8

**Published:** 2023-03-21

**Authors:** Qiongmin Zhang, Zhiqun Yao, Fang Chen, Xue Wang, Man Wang, Junze Lu, Yu Meng, Lei Xu, Yuechen Han, Wenwen Liu, Haibo Wang

**Affiliations:** 1 Department of Otolaryngology-Head and Neck Surgery, Shandong Provincial ENT Hospital, Shandong University, Jinan, Shandong China; 2 Shandong Institute of Otorhinolaryngology, Jinan, Shandong China

**Keywords:** Cochlear hair cell, TIGAR, Teicoplanin, Apoptosis, ROS/P38 MAPK signaling

## Abstract

Teicoplanin is a glycopeptide antibiotic used to treat severe staphylococcal infections. It has been claimed that teicoplanin possesses ototoxic potential, although its toxic effects on cochlear hair cells (HCs) remain unknown. The TP53-induced glycolysis and apoptosis regulator (TIGAR) plays a crucial role in promoting cell survival. Prior research has demonstrated that TIGAR protects spiral ganglion neurons against cisplatin damage. However, the significance of TIGAR in damage to mammalian HCs has not yet been investigated. In this study, firstly, we discovered that teicoplanin caused dose-dependent cell death *in vitro* in both HEI-OC1 cells and cochlear HCs. Next, we discovered that HCs and HEI-OC1 cells treated with teicoplanin exhibited a dramatically decrease in TIGAR expression. To investigate the involvement of TIGAR in inner ear injury caused by teicoplanin, the expression of TIGAR was either upregulated via recombinant adenovirus or downregulated by shRNA in HEI-OC1 cells. Overexpression of TIGAR increased cell viability, decreased apoptosis, and decreased intracellular reactive oxygen species (ROS) level, whereas downregulation of TIGAR decreased cell viability, exacerbated apoptosis, and elevated ROS level following teicoplanin injury. Finally, antioxidant therapy with N-acetyl-L-cysteine decreased ROS level, prevented cell death, and restored p38/phosphorylation-p38 expression levels in HEI-OC1 cells injured by teicoplanin. This study demonstrates that TIGAR may be a promising novel target for the prevention of teicoplanin-induced ototoxicity.

**Supplementary Information:**

The online version contains supplementary material available at 10.1007/s12035-023-03309-8.

## Introduction

Sensorineural hearing loss (SNHL) is the most prevalent sensory disorder, not only affects the quality of life of those afflicted, but also lays a substantial social and economic burden on their families. Ototoxic medications can result in irreversible hair cells (HCs) impairment in the inner ear, leading to SNHL after treatment [1, 2]. Until now, ototoxicity induced by aminoglycosides and platinum-based anticancer medicines, primarily cisplatin, has been the primary focus of research. Notably, ototoxicity is also one of the adverse effects of extensively used glycopeptide antibiotics [3, 4] in clinical settings. Teicoplanin is a glycopeptide antibiotic with a similar chemical structure and antimicrobial spectrum to vancomycin [5, 6]. It is essential for treating serious infections caused by gram-negative bacteria, particularly methicillin-resistant staphylococcus aureus. In addition, teicoplanin is used to treat viral infections caused by influenza virus, hepatitis C virus, etc., and it has been proven to be active *in vitro* against SARS-CoV and recently included on a list of compounds that could be used as part of the therapeutic arsenal against COVID-19 [7]. Due to its low toxicity, teicoplanin may have the potential to replace vancomycin. Teicoplanin has been reported to cause ototoxicity in clinical practice, as administration of teicoplanin to patients with severe infections results in an increase in hearing loss [8–11]. To date, however, the mechanism of teicoplanin ototoxicity has received scant attention, and additional research is required.

TP53-induced glycolysis and apoptosis regulator (TIGAR) is a well-known p53 target protein [12]. TIGAR inhibits intracellular glycolysis by reducing fructose-2,6-bisphosphate levels. Consequently, it facilitates pentose phosphate pathway (PPP) flux to produce nicotinamide adenine dinucleotide phosphate (NADPH) and ribose, thereby promoting DNA repair and decreasing intracellular reactive oxygen species (ROS) [12–14]. TIGAR has numerous essential biological functions, including balancing energy metabolism, protecting mitochondrial function, promoting cell survival, regulating autophagy and stem cell differentiation [15–18]. Recent studies have demonstrated that TIGAR plays a crucial role in variety of diseases, such as cardiovascular diseases, neurological disorders, and malignancies [18–20]. Previously, we have revealed that TIGAR is the effector of Wnt signaling in the auditory system and that it protects cochlear spiral ganglion neurons from ROS accumulation and cisplatin-induced apoptosis [21]. However, the expression and function of TIGAR in sensory epithelium cells and cochlear HCs has not yet been described.

The purpose of this study was to determine the ototoxicity of teicoplanin in cochlear HCs and HEI-OC1 cells, a HC-like cell line, as well as the protective effect of TIGAR on teicoplanin-induced damage in HEI-OC1 cells, with a focus on the possible relationship between TIGAR and ROS/P38 mitogen-activated protein kinase (MAPK) signaling pathway.

## Materials and Methods

### Mouse Cochlear Organotypic Culture

The cochlear organotypic culturing process was performed as described in the previous studies [22, 23]. Wild-type C57BL/6 mice were decapitated at postnatal day (P) 3 after anesthesia, the skulls were opened along the sagittal suture and the temporal bones of both sides were dissected out and placed into 4℃ cold sterile Hank’s Balanced Salt Solution (Hyclone, USA). After removing the cochlear capsule, the stria vascularis and the modiolus, the cochlear basilar membrane was exposed under a dissecting microscope. Cochlear basilar membrane was placed on glass coverslips (Fisher Scientific, PA) coated with CellTaK (BD Biosciences, USA) and incubated in DMEM/F12 (Invitrogen, USA) supplemented with 10% fetal bovine serum (FBS; Gibco, USA), B27(1:50 dilution; Invitrogen), N2 (1:100 dilution, Invitrogen), and ampicillin (50 mg/ml, Sigma) at 37 °C in a 5% CO_2_ atmosphere. The following day, cochlea explants were treated with various concentrations of teicoplanin (0 mM, 3.7 mM, 7.5 mM, and 15 mM) for 24 h to detect HC loss or treated with 7.5 mM for 24 h to detect HC apoptosis.

All animal procedures were performed followed protocols approved by the Animal Care Committee of Shandong University, China (No. ECAESDUSM 20,123,011) and were in accordance with the National Institutes of Health’s Guide for the Care and Use of Laboratory Animals.

### HEI-OC1 Cell Culture

HEI-OC1 cells were cultured at 33 °C with 5% CO_2_ in high-glucose Dulbecco’s Modified Eagle’s Medium (DMEM; Gibco, USA) containing 10% FBS and 50 mg/ml ampicillin [24]. Cell subculture was performed at a density of 1 × 10^5^ cells/ml or 80% confluence using 0.25% trypsin/EDTA (Gibco, USA).

### Detection of Cell Viability and Measurement of the IC50 Value

HEI-OC1 cells at 60% confluence in a 96-well plate were treated with teicoplanin at concentrations of 0 mM, 3 mM, 6 mM, 12 mM, 24 mM for 24 h, then 10 μL CCK-8 reagent (Sigma-Aldrich, USA) was added to each well and incubated for additional 2 h. The absorbance at 450 nm was measured using ELISA reader (Multiskan MK3) for cell viability detection. The IC50 value of the inhibition curve was determined by fitting the inhibition curve to the data using nonlinear regression analysis to generate a four parameters sigmoid dose–response equation (GraphPad Prism, version 9.2.0).

### Immunostaining

After culture, cochlea explants or HEI-OC1 cells on coverslips were fixed with 4% paraformaldehyde and blocked for 1 h at room temperature with PBT-1 (5% donkey serum, 0.1% Triton X-100, 1% bovine serum albumin and 0.02% sodium azide in PBS). The samples were then incubated overnight at 4℃ with primary antibodies diluted in blocking solution against cleaved-Caspase 3 (1:500 dilution; Cell Signaling Technology) and TIGAR (1:800 dilution; Abcam). The next day, the samples were incubated with secondary fluorescent antibodies (1:1000 dilution, Invitrogen) along with DAPI (1:1000 dilution, Sigma-Aldrich) in 0.1% Triton X-100 and 1% bovine serum albumin in PBS at room temperature for 1 h. The coverslips were mounted and imaged using a confocal laser scanning microscope (Leica SP8; Leica, Germany).

For the staining of phalloidin, after fixation, the basilar membranes on coverslips were incubated with iFluor™ 488 phalloidin (1:1000 dilution; Yesen) in PBS along with DAPI (1:1000 dilution) for 30 min in the dark. The coverslips were then mounted and confocal microscopy images were acquired.

### Terminal Deoxynucleotidyl Transferase-Mediated dUTP Nick End-Labeling (TUNEL) Assay

According to the manufacturer’s instructions, the TUNEL assay was employed to measure apoptosis in HEI-OC1 cells and cochlea culture explants (Click-iT Plus TUNEL Assay for In Situ Apoptosis Detection; Invitrogen). DAPI (1:1000 dilution) was used to label all HEI-OC1 cells in the dark, while iFluor™ 488 phalloidin (1:1000 dilution; Yesen) in PBS for 30 min to visualize fluorescently labelled HCs. The coverslips were mounted and imaged using a laser scanning confocal microscope (Leica SP8; Leica, Germany) following treatment.

### RNA Extraction and qRT-PCR

According to the manufacturer’s instructions, total RNA was extracted from mouse cochleae and HEI-OC1 cells using TRIzol (Life Technologies, USA). The total RNA (1 μg) reverse-transcribed to the cDNA using the Revert Aid First Strand cDNA Synthesis Kit (Thermo scientific, USA) according to the manufacturer’s instructions. Quantitative real-time PCR (RT-PCR) was performed to examine the expression of genes using SYBR Premix Ex Taq (TaKaRa, Japan) with *Gapdh* as the housekeeping gene. All data were analyzed using the Eppendorf Realplex 2. PCR primers for the genes are listed in Table 1.

**Table 1:** PCR primer sequences used in the experiment

Gene	Forward sequence	Reverse sequence
Casp3	GGAGCAGCTTTGTGTGTGTG	CTTTCCAGTCAGACTCCGGC
Bcl-2	TGACTTCTCTCGTCGCTACCG	GTGAAGGGCGTCAGGTGCAG
Bax	CGTGGTTGCCCTCTTCTACT	TTGGATCCAGACAAGCAGCC
GAPDH	AGGTCGGTGTGAACGGATTTG	TGTAGACCATGTAGTTGAGGTCA

### Protein Extraction and Western Blot Analysis

The cochlear explants and HEI-OC1 cells were harvested and lysed with radio-immunoprecipitation assay lysis buffer (Protein Biotechnology, China) containing a protease inhibitor cocktail (Sigma, USA) for 30 min on ice. The lysates were centrifuged at 12,000 × *g* for 10 min at 4 °C. The supernatant was collected, and the protein concentrations were measured by the BCA assay (P0012, Beyotime Institute of Biotechnology). Equal amounts of each protein sample were separated by 12% SDS-PAGE and transferred onto polyvinylidene difluoride membranes (Millipore, Germany). The membranes were blocked with 5% non-fat dried milk in Tris-buffered saline and Tween 20 (TBST) for 1 h at room temperature and then incubated with the primary antibodies in TBST containing 3% non‐fat dried milk at 4 °C overnight. The primary antibodies were anti-TIGAR (1:1000 dilution, Abcam), anti‐cleaved Caspase‐3 (1:500 dilution, CST), anti‐Bax (1:500 dilution, Abcam), anti-Bcl-2 (1:500 dilution, Abcam), anti-p38(1:1000 dilution, CST), anti-p-p38(1:2000 dilution, CST) and anti‐β‐actin (1:1000 dilution; ZSGB‐BIO). The protein signals were visualized using an ECL kit (Millipore, Billerica, MA, USA). Semi-quantification of the western blot results was performed using Image J to measure the intensities of the bands.

### Measurement of Mitochondrial Membrane Potential (Δψm)

The changes in mitochondrial membrane potential (Δψm) of HEI-OC1 were tested with Δψm assay kit with JC-1 following the manufacturer’s instruction (Beyotimebio, China). Briefly, HEI-OC1 cells were seeded in 6-well plates at a density of 1 × 10^5^ cells/ml and cultured for 24h. After different drug treatments as described in the text, cells were incubated with JC-1 staining solution in 37 °C for 20 min and washed twice with JC-1 staining buffer (1X). Then cells were stained with DAPI for 10 min at room temperature and photographed by confocal microscope. Finally, the fluorescence images were analyzed by Image J software.

### Adenovirus Administration in HEI-OC1 Cells

The construction of adenovirus (Ad) that overexpressed TIGAR (Ad-TIGAR) was reported in our previous study [21], crude virus was purified using the Adeno-X Virus Purification Kit (BD Biosciences, Clontech), and stored at a concentration of 5 × 10^10^ PFU/mL at -80 ℃. The adenovirus vector conjugated with GFP (Ad-GFP) was used as the negative control. HEI-OC1 cells were seeded in 6- or 96-well plates at a density of 1 × 10^5^ cells/ml and cultured for 24 h. Ad-TIGAR or Ad-GFP diluted 1:1000 in enhanced infection solution (EIS) were added to cells and incubated for 24 h, then the transfection medium were change into normal medium with or without 7.5 mM teicoplanin and cells were harvest for further analysis after incubation for 24 h.

### shRNA Transfection in HEI-OC1 Cells

The TIGAR-specific shRNA (shRNA-TIGAR) was designed and synthesized (GenePharma, China) to knock down the mRNA expression of TIGAR in HEI-OC1 cells. The negative control of shRNA-TIGAR was the plasmid carried a non-targeting sequence (shRNA-GFP). HEI-OC1 cells were seeded in 6- or 96-well plates at a density of 1 × 10.^5^ cells/ml and cultured for 24 h. Lipofectamine™ 3000 Reagent (Thermo Fisher Scientific, USA) diluted in Opti-MEM (Gibco, BRL) was added to the mixture containing DNA (2 µg/µL) and P3000™ enhancer Reagent for 10 min. Then the total DNA-lipid complex was added to the cells. The Opti‐MEM medium was replaced with DMEM containing FBS after 8 h. The cells were further treated with 7.5 mM teicoplanin with or without pretreatment of 2 mM N‐acetylcysteine (NAC; A7250; Sigma, USA) for 2 h, HEI-OC1 cells were harvest for further analysis after incubation at 33 °C and 5% CO_2_ for 24 h. The shRNA sequences were listed in Table 2

**Table 2:** ShRNA sequences used in the experiment

shRNA	sense	antisense
ShRNA-Control	GTTCTCCGAACGTGTCACGT	ACGTGACACGTTCGGAGAAC
shRNA-TIGAR	GGATCAGTGTCTTCATCATAG	CTATGATGAAGACACTGATCC

### Hair Cell Counting and Cochleogram Plotting

The quantitative evaluation of HCs was performed according to our previously published report[22, 23]with slight modification. After the HCs immunostaining with phalloidine, the apical, middle, and basal turns were first imaged under × 20 magnification to identify regions of interest, and at least two Z-stacks from non-overlapping regions were then obtained for each turn using a × 40 objective. Image J software was used to measure the length of the selected cochlear segments, the total number of HCs was counted in each of the three cochlear segments (apex, middle, and base). A cochleogram was constructed to show the percentage of innerHCs (IHCs) and the loss of outer HCs (OHCs) as a function of the percent distance from the apical to the basal turn of the cochlea. The losses of HCs from individual cochleogram were averaged to generate a mean cochleogram for each condition using custom software.

### Statistical Analysis

For each condition, at least three individual experiments were repeated. All data were presented as the mean ± SEM. Statistical analyses were performed using Graphpad Prism 9.0 and SPSS19.0 software. Two-tailed, unpaired Student’s t-tests were used to determine statistical significance when comparing two groups. A one-way ANOVA followed by a Dunnett’s multiple comparisons test was used when comparing more than two groups. A value of p < 0.05 was considered statistically significant.

## Results

### Teicoplanin Induced HEI-OC1 Cell Loss and Cochlear HC Loss in a Dose-Dependent Manner *in vitro*

**Fig. 1:**
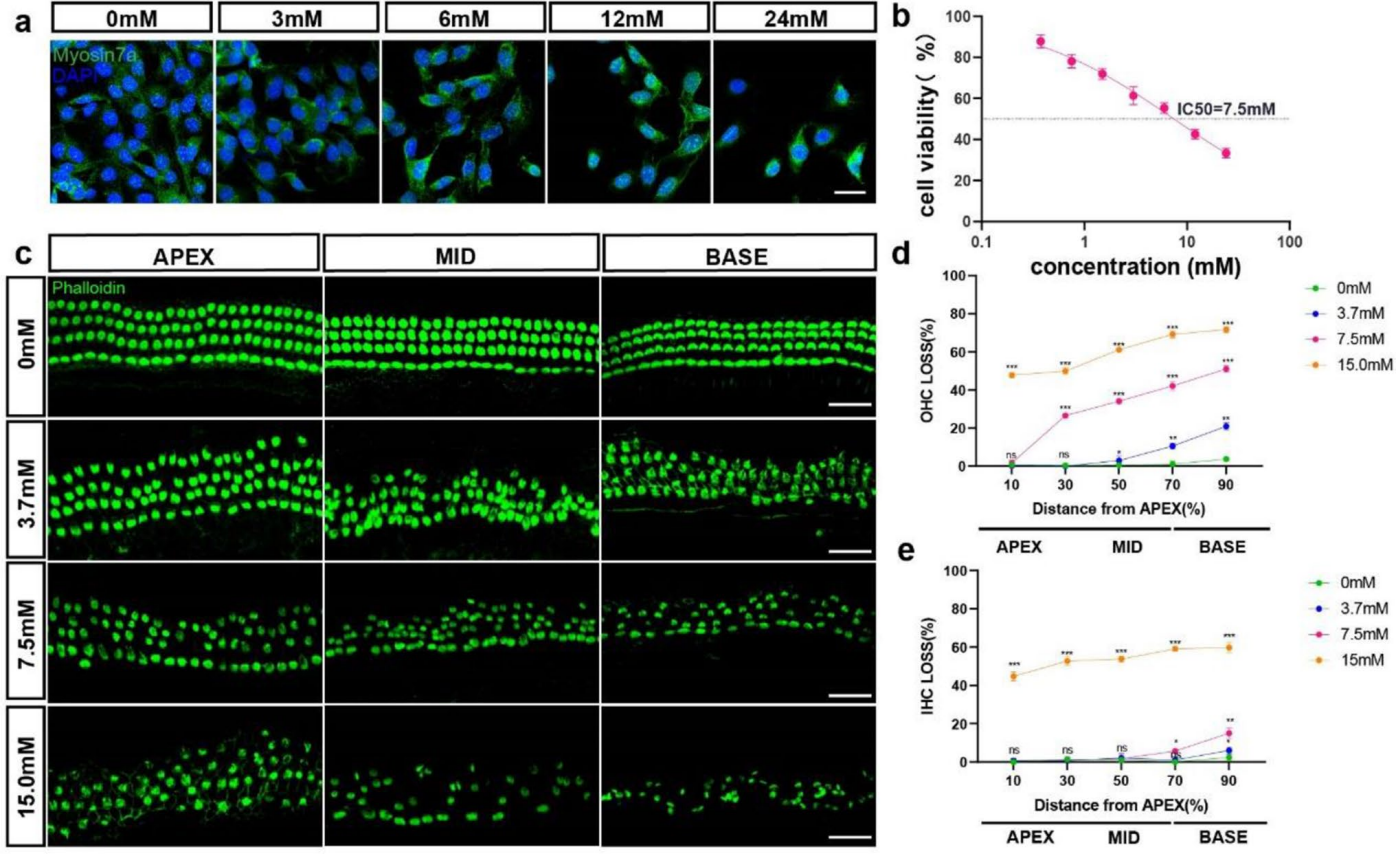
Teicoplanin induced HEI-OC1 cell loss and cochlear HC loss in a dose-dependent manner *in vitro.* **a**. Representative immunostaining images of HEI-OC1 cells labeled with HC marker Myosin 7a (green) and DAPI (blue) after different concentrations (0, 3, 6, 12, 24 mM) of teicoplanin administration for 24 h respectively. **b**. CCK-8 assay showed that the HEI-OC1 cell viability was markedly decreased after teicoplanin administration compared to the control group, and the reduction was in a dose-dependent manner. IC50 curve showed that 7.5 mM teicoplanin was about to cause a 50% cell viability in HEI-OC1 cells. **c**. Representative immunostaining images of cochlear HCs (phalloidin, green) treated with teicoplanin (0, 3.7, 7.5, 15.0 mM) for 24 h respectively *in vitro.* **d**, **e**. The mean cochleogram showed that teicoplanin caused a basal–apical gradient of HC loss and both IHC and OHC losses were exacerbated with teicoplanin dose increasing. * *P* < 0.05, ** *P* < 0.01, *** *P* < 0.001. Scale bar = 20 μm

HEI-OC1 cells were subjected to various concentrations of teicoplanin (0, 3, 6, 12, 24 mM) for 24 h to determine the possible cytotoxicity of teicoplanin on auditory cells. Immunostaining results indicated that HEI-OC1 treated with teicoplanin had a decrease in cell quantity and shrunken nuclei. CCK-8 assay and statistical analysis revealed that the HEI-OC1 cell viability was significantly decreased to 65.57 ± 3.15% after 3 mM teicoplanin administration compared to the control group, and it was further reduced to 61.20 ± 1.90%, 51.93 ± 1.69% and 45.23 ± 1.72% after teicoplanin of 6, 12, 24 mM concentrations, respectively. As the concentration of teicoplanin increase, fewer HEI-OC1 cells were able to survive, indicating that the reduction in cell viability was dose-dependent (Fig. 1a, b). According to the IC50 curve, 7.5 mM teicoplanin was about to produce a 50% cell viability in HEI-OC1 cells (Fig. 1b), hence, 7.5 mM teicoplanin treatment for 24 h was selected as the treatment condition for the subsequent HEI-OC1 cell experiments.

Next, based on the dosage response assays of HEI-OC1 cells, cultured cochlea HCs were treated with *in vitro* teicoplanin at concentration of 3.7 mM, 7.5 mM, and 15 mM for 24 h respectively*.* Immunostaining and mean cochleograms were utilized to evaluate the location and percentage loss of OHCs and IHCs induced by teicoplanin. As illustrated in Fig. 1c-e, the greatest severe loss of HCs occurred at the base of the cochlea and decreased toward the apex, that is, teicoplanin induced a basal–apical gradient of HC loss. In addition, there was a greater loss of OHCs than IHCs after teicoplanin treatment, and both IHC and OHC losses exacerbated as the teicoplanin dose was increased. Specifically, there were barely any IHCs missing in the apex and middle cochlea turns of the 3.7 mM and 7.5 mM teicoplanin-treated groups, while there were considerable IHC losses in the basal cochlea turns (Fig. 1c, e, Table 3). Treatment with 3.7 mM teicoplanin resulted in modest OHC loss while 7.5 mM teicoplanin led to moderate OHC loss in the middle and basal turns of cochlea, but no significant OHC loss was found in the apical turn in either group (Fig. 1c, e, Table 3). However, 15 mM teicoplanin induced considerable losses of both IHCs and OHCs along the entire length of the cochlea (Fig. 1c-e, Table 3).

**Table 3:** HC loss (%) in mouse cochlea treated with teicoplanin

% Distance from APEX	Cochlea HCs	Control (%)	3.7 mM (%)	7.5 mM (%)	15 mM (%)
10	IHC	0.88 ± 0.38	1.16 ± 0.59	1.70 ± 0.77	45.34 ± 1.35
	OHC	1.02 ± 0.63	1.16 ± 0.59	1.96 ± 0.94	48.52 ± 1.83
30	IHC	1.34 ± 0.52	1.66 ± 0.52	1.66 ± 0.52	52.68 ± 1.45
	OHC	1.24 ± 0.25	1.20 ± 0.37	26.80 ± 1.04	51.14 ± 2.04
50	IHC	1.72 ± 0.30	2.68 ± 0.30	4.00 ± 0.98	54.70 ± 1.81
	OHC	1.10 ± 0.20	5.66 ± 0.26	34.76 ± 1.01	60.80 ± 1.46
70	IHC	1.60 ± 0.20	3.62 ± 0.64	5.96 ± 0.92	59.46 ± 1.05
	OHC	3.52 ± 0.49	10.86 ± 0.88	43.62 ± 2.63	68.74 ± 1.62
90	IHC	2.82 ± 0.27	6.20 ± 1.15	14.26 ± 1.46	60.40 ± 1.99
	OHC	3.82 ± 0.38	21.30 ± 0.89	52.12 ± 1.95	71.96 ± 1.07

### Teicoplanin Induced Apoptosis in HEI-OC1 Cells and Cochlear HCs

**Fig. 2:**
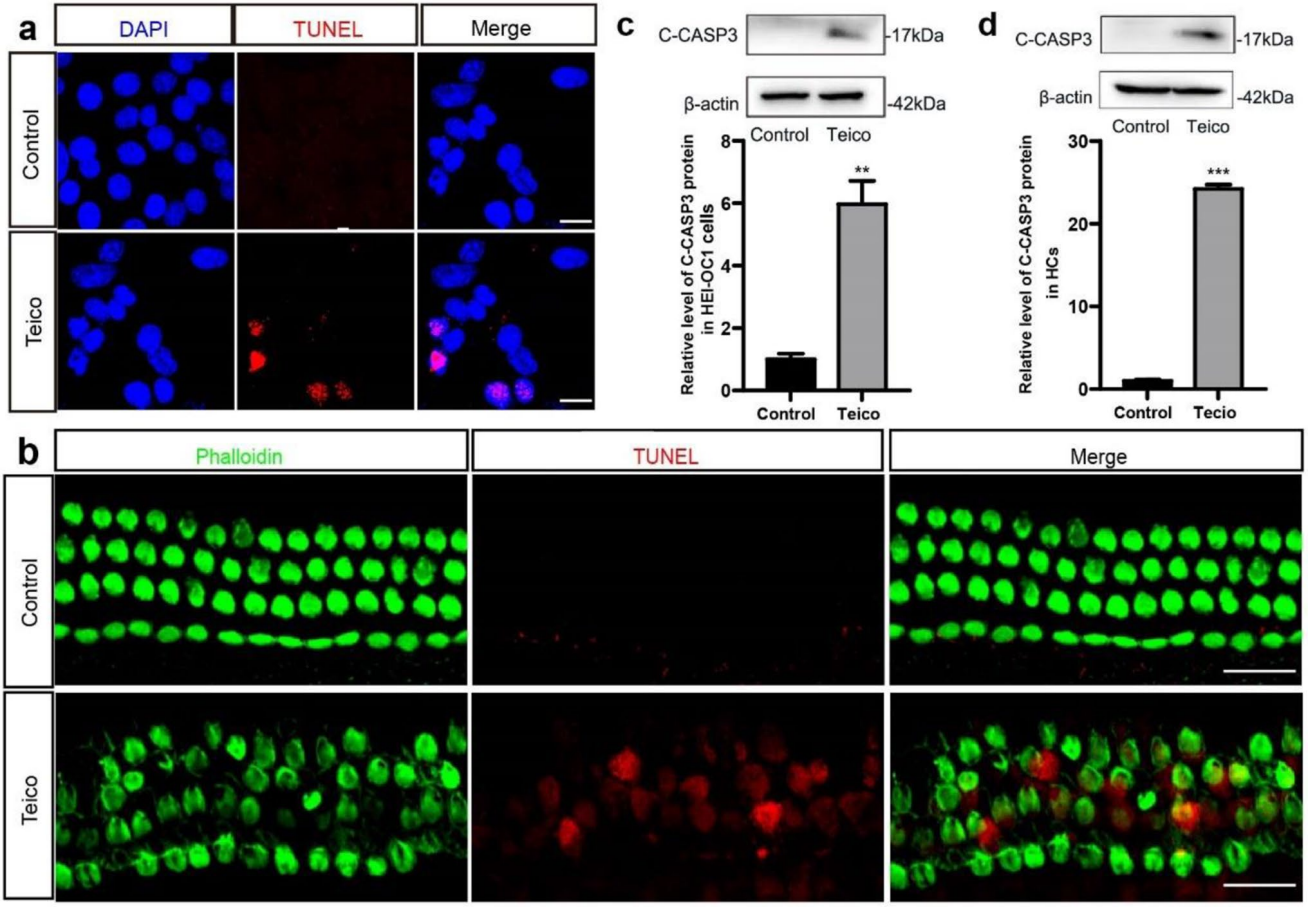
Teicoplanin induced apoptosis in HEI-OC1 cells and cochlear HCs. **a**. TUNEL results showed that there was no TUNEL (red) positive HEI-OC1 cells (DAPI, blue) in the control group while nuclei shrinkage and clear TUNEL positive cells were found in HEI-OC1 cells after 7.5 mM teicoplanin treatment for 24 h. **b**. Representative fluorescence images of cochlear basal turn HCs (phalloidin, green) showed that the cultured cochlear basal turn HCs kept in alignment and no TUNEL (red) positive HCs were detected in the control group, while HCs fell into morphological disorder and many HCs were labelled with TUNEL after exposure to 7.5 mM teicoplanin. **c**, **d**. the protein levels of cleaved Caspase-3 in HEI-OC1 cells and cochlear HCs were both increased significantly after teicoplanin injury, compared to the control groups. ** *P* < 0.01, *** *P* < 0.001. Scale bar = 20 μm

The cellular apoptosis of HEI-OC1 cells and cochlear HCs induced by teicoplanin was assessed using TUNEL assay and cleaved Caspase-3 expression examination. HEI-OC1 cells or cochlea HCs were treated with 7.5 mM teicoplanin for 24 h. TUNEL results revealed that there was no TUNEL-positive HEI-OC1 cells in the control group, but HEI-OC1 cells treated with teicoplanin exhibited nuclei shrinkage and clear TUNEL-positive cells (Fig. 2a). Based on our findings in Fig. 1c that the HCs in the basal turn are the most vulnerable to teicoplanin injury, fluorescence staining results of cochlear basal turn HCs were selected as the representative images. As illustrated in Fig. 2b, the cultured cochlear basal turn HCs remained aligned and no TUNEL-positive HCs were detected in the control group*.* In contrast, HCs exhibited morphological disorder and significant amount of apoptotic HCs, which were cleaved Caspase 3-postive, were observed in the 7.5 mM teicoplanin-treated group (Fig. 2b). Moreover, the protein levels of cleaved Caspase-3 in HEI-OC1 cells and cochlear HCs were both increased significantly after teicoplanin injury, compared to the control groups (Fig. 2c, d). These results indicated that exposure to teicoplanin induced apoptosis in HEI-OC1 cells and cochlear HCs *in vitro*.

### The Expression of TIGAR was Decreased in HEI-OC1 Cells and Cochlear HCs after Teicoplanin Damage

**Fig. 3:**
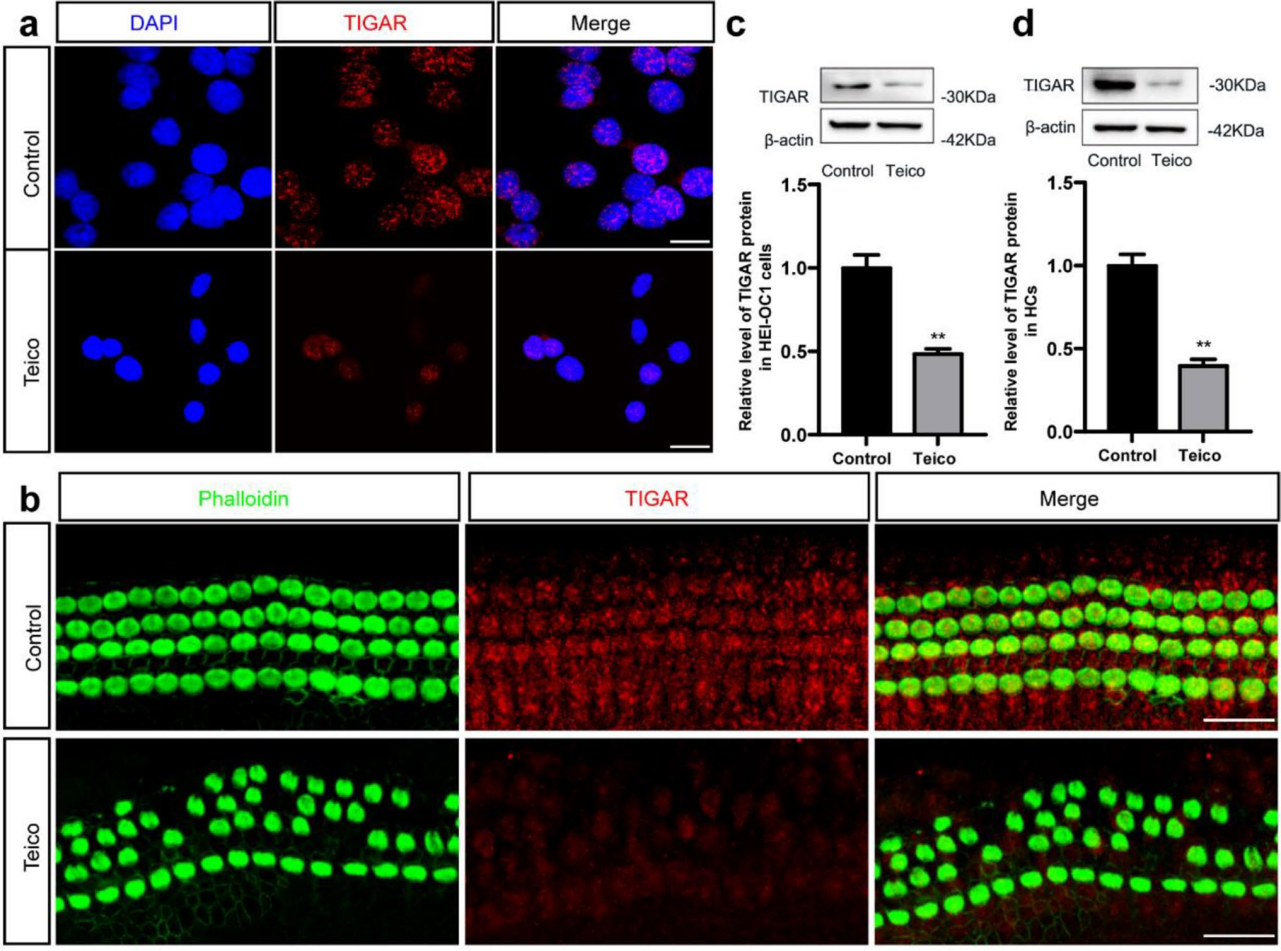
The expression of TIGAR was decreased in HEI-OC1 cells and cochlear HCs after teicoplanin damage. **a**, **b**. HEI-OC1 cells and cochlear HCs were treated with 7.5 mM teicoplanin for 24 h respectively. Immunostaining results revealed that the fluorescence intensity of TIGAR (red) was markedly reduced in survived HEI-OC1 cells (DAPI, blue) and cochlear HCs (phalloidin, green) after teicoplanin treatment in contrast to the undamaged controls. **c**, **d**. Western blot results verified that the expression levels of TIGAR in HEI-OC1 cells and cultured cochlear HCs were both significantly decreased after teicoplanin administration compared to the control groups. ** *P* < 0.01. Scale bar = 20 μm

The expression of TIGAR in postnatal cochlea HCs in the inner ears of C57BL/6 mice of varying ages (P1, 3, 7, 14, 30) was described. Immunostaining results showed that TIGAR was clearly expressed in all three turns of cochlea HCs from P1 to P30. Middle turn HCs were selected as the typical samples, and their images were illustrated in supplementary Fig. 1. Next, to determine whether the TIGAR expression in auditory cells was affected by teicoplanin treatment, HEI-OC1 cells and cochlear HCs were treated with 7.5 mM teicoplanin for 24 h, and then the changes in TIGAR expression were detected*.* As shown in Fig. 3, immunostaining results revealed that the fluorescence intensity of TIGAR was markedly reduced in survived HEI-OC1 cells (Fig. 3a) and cochlear HCs (Fig. 3b) after teicoplanin treatment in contrast to the undamaged controls. Furthermore, western blot results verified that the expression levels of TIGAR in HEI-OC1 cells and cultured cochlear HCs were considerably lower in the teicoplanin-treated groups compared to the control groups (Fig. 3c, d). Together, these results demonstrated that teicoplanin injury led to a reduction of TIGAR expression in HEI-OC1 cells and HCs, indicating a link between teicoplanin-induced ototoxicity and TIGAR expression.

### Knockdown of TIGAR Expression Decreased HEI-OC1 Cell Viability while the Overexpression of TIGAR Increased it after Teicoplanin Injury

**Fig. 4:**
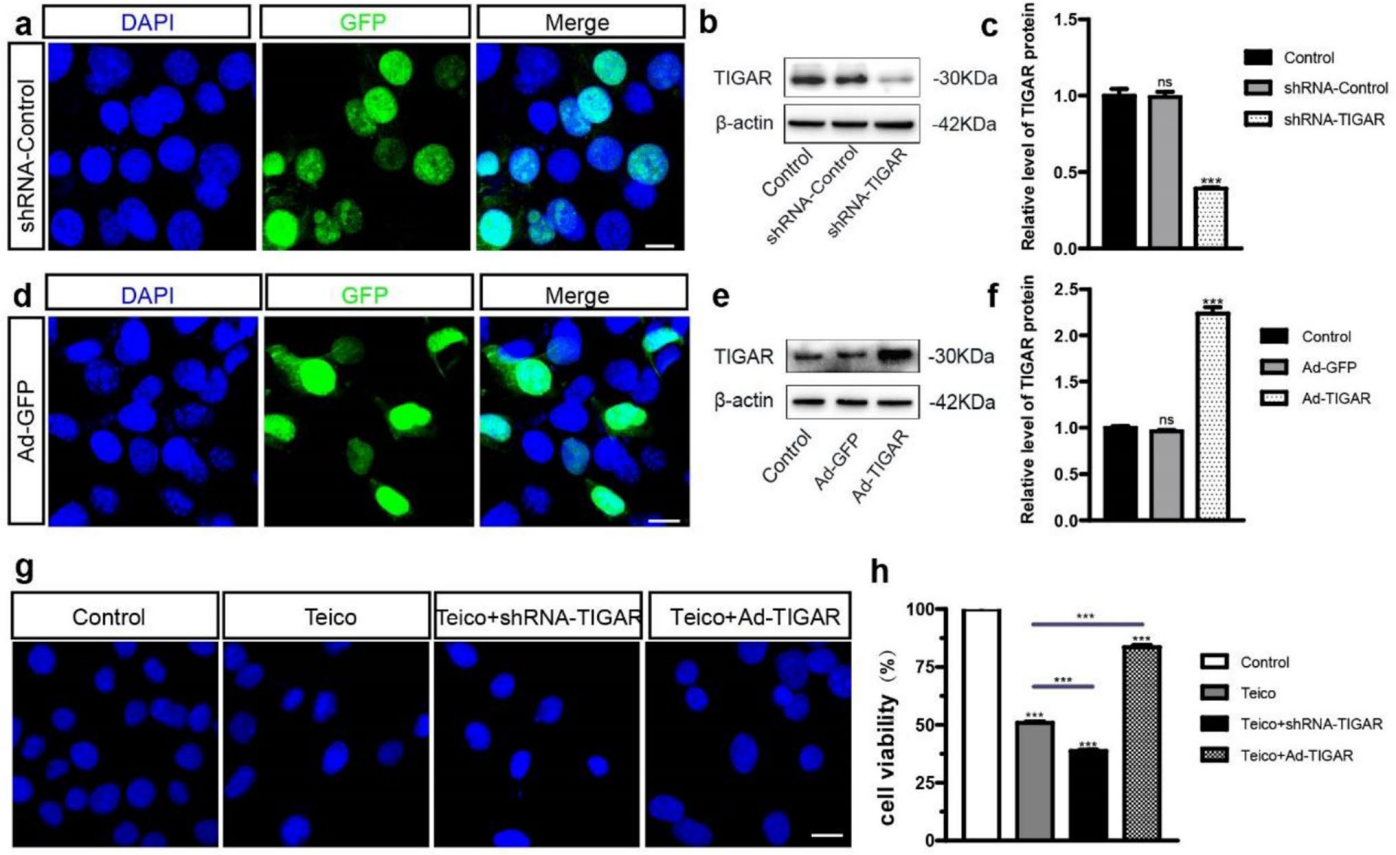
Knockdown of TIGAR expression decreased HEI-OC1 cell viability while the overexpression of TIGAR increased it after teicoplanin injury. **a**. Immunofluorescence staining results showed a successful shRNA transfection in HEI-OC1 cells as indicated by the co-label of GFP and DAPI. **b**, **c**. Western blot showed that protein level of TIGAR was significantly decreased in shRNA-TIGAR group of HEI-OC1 cells, but it was remained unchanged in shRNA-Control group compared to the normal control group. **d**. The efficiency of the virus infection was measured using empty adenovirus vector conjugated with GFP (Ad-GFP), and immunostaining result verified the effective infection via GFP and DAPI co-labelling. **e**, **f**. Western blot showed that the level of TIGAR in Ad-TIGAR‐treated cells was significantly upregulated while no change in AD-GFP group was found compared with control group. **g**. Immunostaining illustrated an obvious cell number reduction and nuclei shrunk in the Teico + shRNA-TIGAR group, whereas an increase of cell number in Teico + Ad-TIGAR group compared to the teicoplanin only group. **h**. The CCK-8 assay showed that the HEI-OC1 cell viability was further reduced in the Teico + shRNA-TIGAR group, while the cell viability was increased in Teico + Ad-TIGAR group in contrast to the teicoplanin group. *** *P* < 0.001. Scale bar = 20 μm

To further investigate the role of TIGAR in teicoplanin-induced damage to auditory cells, HEI-OC1 cells were transfected with shRNA or recombinant adenovirus to downregulate or upregulate TIGAR expression, respectively. The efficiency of shRNA transfection was determined. The control group consisted of untreated HEI-OC1 cells, the shRNA-Control group consisted of HEI-OC1 cells transfected with nonsense shRNA conjugated with GFP (shRNA-GFP), and the shRNA-TIGAR group consist of cells transfected with TIGAR-specific shRNA. Immunofluorescence staining results showed a successful shRNA transfection as indicated by the co-label of GFP and DAPI (Fig. 4a). Western blot showed that protein level of TIGAR was dramatically decreased in shRNA-TIGAR group compared to the normal control group, but remained unchanged in shRNA-Control group (Fig. 4b, c). The efficiency of the virus infection was measured using empty adenovirus vector conjugated with GFP (Ad-GFP), and immunostaining result verified the effective infection via GFP and DAPI co-labelling (Fig. 4d). Western blot showed that the level of TIGAR in Ad-TIGAR-treated cells was significantly upregulated, whereas no change was observed in AD-GFP group compare to the control group (Fig. 4e, f). These results demonstrated that the expression of TIGAR was efficiently knocked down by shRNA-TIGAR while it was overexpressed via Ad-TIGAR in HEI-OC1 cells.

We then examined the effect of TIGAR on the viability of HEI-OC1 cells after teicoplanin administration. 24 h after pretreatment with either shRNA-TIGAR or Ad-TIGAR, HEI-OC1 cells were co-treated with 7.5 mM teicoplanin. Immunostaining and CCK-8 assay revealed that the HEI-OC1 cell viability was further reduced in the Teico + shRNA-TIGAR group (38.78 ± 1.36%), whereas it was increased in Teico + Ad-TIGAR group (83.64 ± 2.41%), in contrast to the teicoplanin group (50.92 ± 1.72%), indicating that TIGAR inhibition makes the HEI-OC1 cells more sensitive to teicoplanin-induced cell death and the overexpression of TIGAR protects the survival of HEI-OC1 cells (Fig. 4g, h).

### TIGAR Deficiency Aggravated HEI-OC1 Cell Apoptosis but TIGAR Overexpression Protected HEI-OC1 Cells from Apoptosis after Teicoplanin Injury

**Fig. 5:**
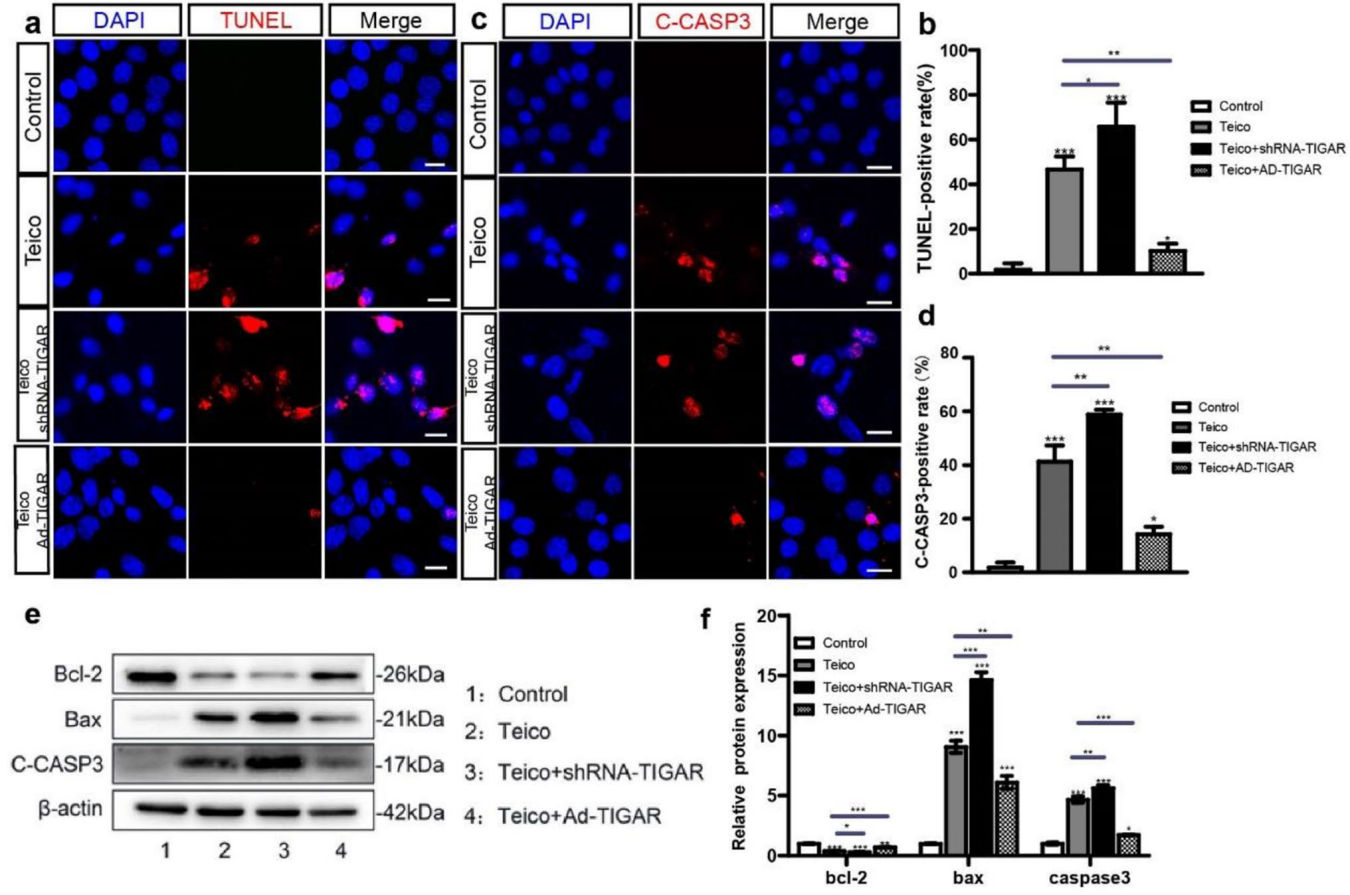
TIGAR deficiency aggravated HEI-OC1 cell apoptosis but TIGAR overexpression protected HEI-OC1 cells from apoptosis after teicoplanin injury. **a**-**d**. Apoptotic cells exhibited TUNEL-positive fluorescence (red) or cleaved-caspase3-positive fluorescence (red) after treatment with teicoplanin, and the numbers of TUNEL-positive cells or cleaved-caspase3-positive cells in the Teico + shRNA-TIGAR group were increased significantly whereas were decreased in the Teico + Ad-TIGAR group compared to the Teico group. **e, f**. Western bolt results showed that teicoplanin increased the protein expression of Bax and cleaved Caspase-3, while it reduced the expression of Bcl-2, compared to the control group. The decreases in the levels of Bcl-2 and increases in the levels of Bax and cleaved Caspase-3 were reversed by TIGAR overexpression, whereas they were exacerbated by TIGAR knockdown, in HEI-OC1 cells after teicoplanin treatment. * *P* < 0.05, ** *P* < 0.01, *** *P* < 0.001. Scale bar = 20 μm

The TUNEL assay and cleaved-Caspase 3 immunostaining were performed to determine apoptosis in HEI-OC1 following co-treatment with teicoplanin or TIGAR deficiency or overexpression. Apoptotic cells exhibited TUNEL-positive fluorescence (Fig. 5a) or cleaved-caspase3-positive fluorescence (Fig. 5c) after treatment with teicoplanin, and the numbers of TUNEL-positive cells or cleaved-caspase3-positive cells were significantly increased in Teico + shRNA-TIGAR group but decreased in Teico + Ad-TIGAR group (Fig. 5b, d).

We further investigated the potential mechanism underlying the apoptotic response by evaluating the activity of apoptosis-related genes. HEI-OC1 cells treated with 7.5 mM teicoplanin for 24 h exhibited considerably increased expression of pro-apoptotic genes Bax and cleaved Caspase-3, but dramatically decreased expression of anti-apoptotic gene Bcl-2, compared to the control group (Fig. 5e). TIGAR overexpression reversed the declines in Bcl-2 and increases in Bax and cleaved Caspase-3 that were caused by teicoplanin administration in HEI-OC1 cells, whereas TIGAR knockdown exacerbated these changes (Fig. 5e). These results suggested that TIGAR overexpression protects HEI-OC1 cells from apoptosis, while TIGAR knockdown aggravated teicoplanin-induced cell apoptosis.

### TIGAR Overexpression Decreased the Generation of ROS, while TIGAR Knockdown Exacerbated the Accumulation of ROS in HEI-OC1 Cells after Teicoplanin Damage

**Fig. 6:**
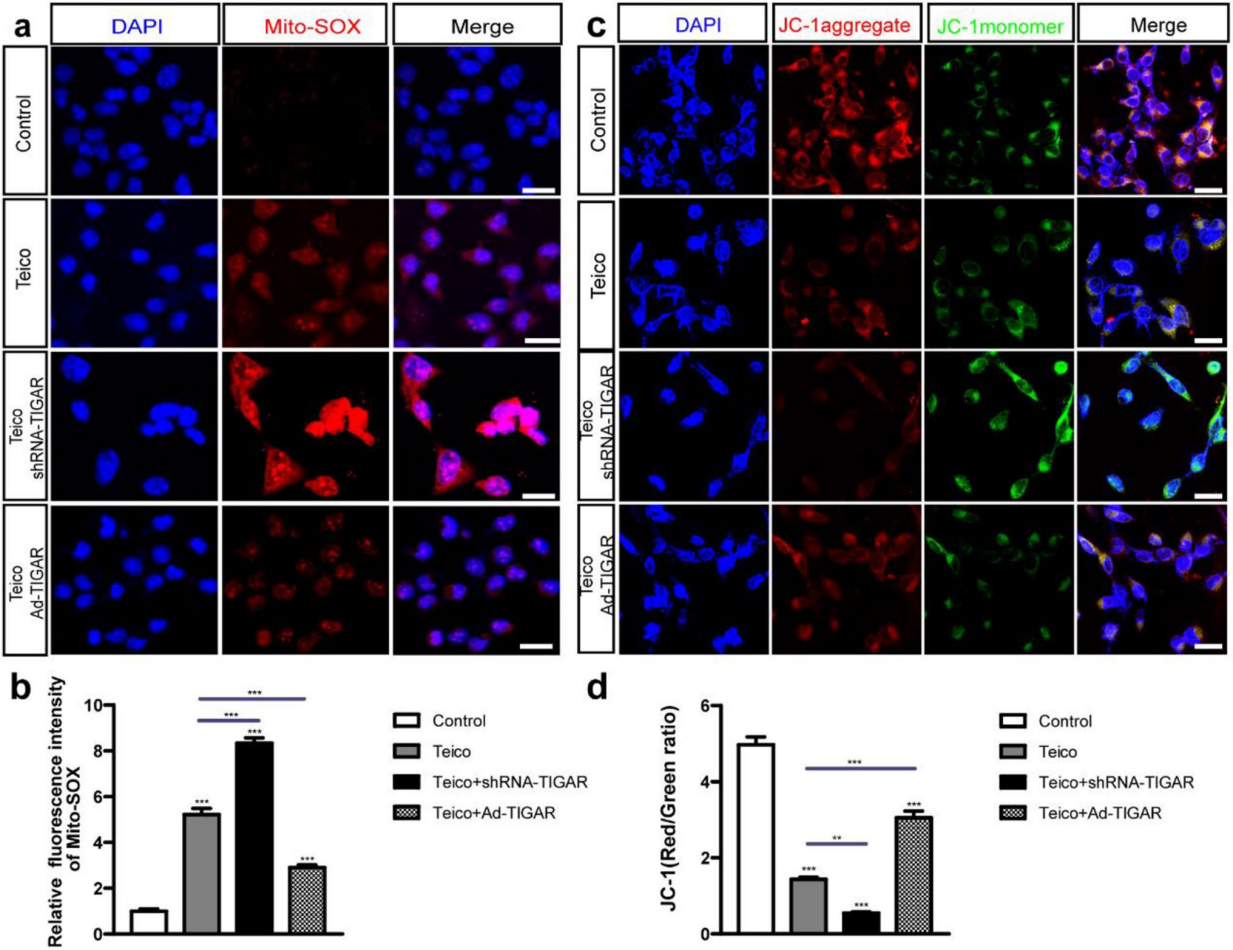
TIGAR overexpression decreased the generation of ROS, while TIGAR knockdown exacerbated the accumulation. **a, b**. The relative fluorescence intensity of Mito-SOX was significantly upregulated after teicoplanin treatment compared to that in control HEI-OC1 cells. The pretreatment with shRNA-TIGAR transfection enhanced the fluorescence intensity further, while pretreatment with adenovirus infection of TIGAR reduced the fluorescence intensity compared to teicoplanin group. **c**,** d**. Immunostaining results showed that JC-1 fluorescence (red/green ratio) was reduced significantly after teicoplanin treatment compared to the control group, and the JC-1 fluorescence (red/green ratio) in Teico + shRNA-TIGAR group was further reduced, whereas it was raised significantly in Teico + Ad-TIGAR group in contrast to the Teico group. ** *P* < 0.01, *** *P* < 0.001. Scale bar = 20 μm

It has been established that ROS a close association with the process of HC damage induced by ototoxic drugs [25–27]. To determine the relationship between teicoplanin and oxidative stress in HEI-OC1 cells, the Mito-SOX Red was utilized to evaluate mitochondrial ROS accumulation in HEI-OC1 cells after treatment with teicoplanin and the regulation of TIGAR expression. HEI-OC1 cells were incubated with either shRNA-TIGAR or Ad-TIGAR before the co-treatment with 7.5 mM teicoplanin for 24 h. Immunostaining results demonstrated that the relative fluorescence intensity of Mito-SOX red was significantly upregulated in HEI-OC1 cells treated with teicoplanin compared to control cells (Fig. 6a, b). Pretreatment with shRNA-TIGAR transfection enhanced the fluorescence intensity further, while pretreatment with Ad-TIGAR decreased the fluorescence intensity relative to teicoplanin group (Fig. 6a, b). The accumulation of ROS can induce mitochondria oxidative stress injury. Therefore, we assess the mitochondrial membrane potential (ΔΨm) of HEI-OC1 cells with various pharmacological treatments using the JC-1 mitochondrial staining test. JC-1 aggregates fluorescence (red) accumulated in the mitochondrial membrane emitted a stronger fluorescent signal than monomeric JC-1 (green) in control HEI-OC1 cells, but the monomeric JC-1 signals were increased after teicoplanin injury, indicating the depolarization of the mitochondrial membrane (Fig. 6c). The ratio of JC-1 fluorescence (red/green ratio) was utilized to evaluate the change of the ΔΨm. As shown in Fig. 6d, JC-1 fluorescence was significantly reduced after teicoplanin treatment compared to the control group, and it was further decreased in Teico + shRNA-TIGAR group, whereas it was significantly increased in the Teico + Ad-TIGAR group compare to the Teico group. These results demonstrated that TIGAR was effective at inhibiting the accumulation of ROS in HEI-OC1 cells exposed to teicoplanin, which was directly associated to mitochondrial membrane depolarization.

### Antioxidant Treatment with NAC Lowered ROS Level, Rescued HEI-OC1 Cell Loss and Apoptosis as well as Restored p38/p-p38 Expression Levels Induced by TIGAR Deficiency after Teicoplanin Injury

**Fig. 7:**
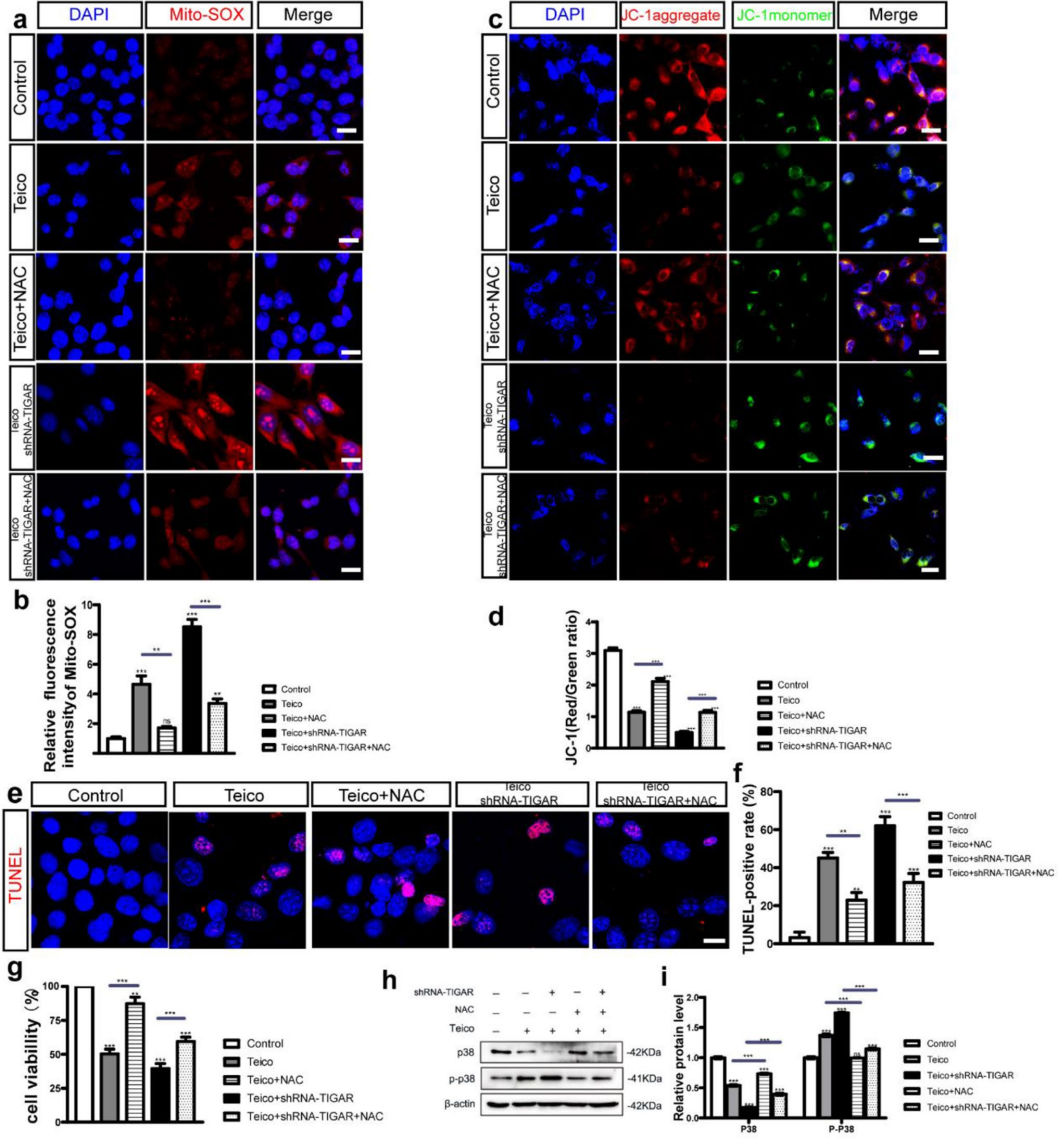
Antioxidant treatment with NAC lowered ROS level, rescued HEI-OC1 cell loss and apoptosis as well as restored p38/p-p38 expression levels induced by TIGAR deficiency after teicoplanin injury. **a, b**. The relative fluorescence intensity of Mito-SOX was reduced significantly in Teico + NAC group compared to the teicoplanin group, and in Teico + shRNA-TIGAR + NAC group in contrast to Teico + shRNA-TIGAR group after NAC treatment. **c**,** d**. Immunostaining results showed that JC-1 fluorescence (red/green ratio) in groups with NAC treatments had a significant increase compared to their control groups in HEI-OC1 cells after teicoplanin treatment. **e**,** f**. Representative images showed that apoptotic cells exhibited TUNEL-positive fluorescence (red) after treatment with NAC were reduced in Teico + NAC group compared to the teicoplanin group, and they were also decreased in Teico + shRNA-TIGAR + NAC group compared to Teico + shRNA-TIGAR group. **g**. The CCK-8 assay showed that the number of surviving HEI-OC1 cells in Teico + NAC group was significantly increased compared to the teicoplanin only group, as well as in Teico + shRNA-TIGAR + NAC group versus Teico + shRNA-TIGAR group. **h, i**. The western bolt results demonstrated P38 expression level was reduced while p-P38 expression level was increased in HEI-OC1 cells after teicoplanin injury. After treatment with NAC, P38 protein level was increased and the p-P38 expression was decreased in Teico + NAC group compared to Teico group, as well as in Teico + shRNA-TIGAR + NAC group in contrast to Teico + shRNA-TIGAR group. β-actin served as control. **p < 0.01, ***p < 0.001. Scale bar = 20 μm

To further investigate the effect of TIGAR on suppressing ROS accumulation in HEI-OC1 cells induced by teicoplanin, a rescue experiment was performed using the ROS scavenger NAC in TIGAR-deficient HEI-OC1 cells following teicoplanin-induced injury. The dose of NAC was chosen according to our published studies [21, 28], and the result of NAC dose responses which showed that 2 mM NAC pre-treatment successfully rescued the HEI-OC1 cell loss from teicoplanin damage (Supplementary Fig. 2). Then the HEI-OC1 cells were pre-treated with 2 mM of NAC for 2 h before the co-treatment of 7.5 mM teicoplanin and shRNA-TIGAR. The accumulation of ROS was detected by the intensified immunostaining signals of Mito-SOX Red in HEI-OC1 cells. The relative fluorescence intensity of Mito-SOX was reduced significantly in the Teico + NAC group compared to the teicoplanin group, and in the Teico + shRNA-TIGAR + NAC group compare to the Teico + shRNA-TIGAR group (Fig. 7a, b). Moreover, the analysis of JC-1 fluorescence staining demonstrated that JC-1 fluorescence (red/green ratio) in HEI-OC1 cells treated with teicoplanin was significantly increased in NAC-treated groups compared to control groups (Fig. 7c, d).

We then detected the survival and apoptosis of HEI-OC1 cells with or without NAC treatment. TUNEL assay revealed that the TUNEL-positive cells were reduced in the Teico + NAC group compared to the teicoplanin group, and they were also reduced in the Teico + shRNA-TIGAR + NAC group compared to the Teico + shRNA-TIGAR group (Fig. 7e, f). The NAC treatment significantly increased the number of surviving HEI-OC1 cells in the Teico + NAC group (87.44 ± 8.10%) compared to the teicoplanin-only group (50.50 ± 3.41%), as well as in the Teico + shRNA-TIGAR + NAC group (59.55 ± 3.17%) versus the Teico + shRNA-TIGAR group (39.62 ± 3.56%) (Fig. 7g).

To further explore the potential underlying mechanisms of regulating ROS accumulation in HEI-OC1 cells, we examined the P38 MAPK signaling pathway, which is involved in oxidative stress-induced cell apoptosis [29]. The expression and phosphorylation of p38 were identified in HEI-OC1 cells treated with teicoplanin and TIGAR knockdown. As illustrated in Fig. 7h, TIGAR knockdown aggravated the teicoplanin-induced decrease in p38 expression and rise in p-p38 in HEI-OC1 cells. In contrast, a significant increase of p38 protein level and a decrease in p38 phosphorylation level were observed in Teico + NAC group compared to the Teico group, as well as in Teico + shRNA-TIGAR + NAC group compare to Teico + shRNA-TIGAR group (Fig. 7h).

Collectively, our observations revealed that the antioxidant treatment with NAC successfully reduced the ROS accumulation, reversed the increased apoptosis and cell loss in HEI-OC1 cells induced by TIGAR knockdown following teicoplanin injury, and prevented the formation of ROS. In addition, the data suggested that teicoplanin exposure might activate the ROS/P38 signaling pathway in HEI-OC1 cells, and that NAC treatment contributes to restoring the p38/p-p38 expression levels induced by teicoplanin injury and TIGAR deficiency.

## Discussion

Teicoplanin, a glycopeptide antibiotic routinely used to treat Gram-positive bacterial infections, has also shown efficacy against various viruses such as influenza virus, Ebola virus, and COVID-19 [7, 30], thus its clinical application is anticipated to expand in the near future. Teicoplanin has a more favorable safety profile than vancomycin, especially in terms of ototoxicity. The risk of teicoplanin ototoxicity may, however, be underestimated and should be interpreted accordingly [31, 32]. Here, for the first time, we demonstrated that teicoplanin induced considerable cochlea HCs loss and HEI-OC1 cells loss in a dose-dependent manner (Fig. 1), as well as evident cell apoptosis in cultured mouse cochlea HCs and HEI-OC1 cells (Fig. 2). In particular, we found that teicoplanin caused a basal–apical gradient of HC loss, as the most severe loss of HCs was located at the base of the cochlea and decreased toward the apex. This finding is consistent with the clinical report that administration of teicoplanin to patients results in a significant increased hearing loss in high frequencies (4 and 8 kHz) but not in lower frequencies [9]. Consequently, our findings illustrate the cytotoxicity of teicoplanin on auditory cells and reveal a potential adverse effect of its clinical application, particularly as the concentration increases. Since these ototoxicity of aminoglycoside antibiotics and anti-tumor medications has received considerable attention in research, it is crucial to be aware of the ototoxic potential of antibiotics.

TIGAR is an endogenous glycolysis inhibitor and is expressed in nearly all mammalian tissues, with significant abundance in muscle, brain and heart [18, 33]. The expression level of TIGAR gradually decreases with aging, which may be related to the increased susceptibility of neurons to ischemic injury with aging [34]. However, elevated expression of TIGAR has been detected in numerous types of cancers, which may be contributed to its function of promoting cell survival [33, 35]. In this study, we demonstrated that TIGAR was expressed stably in mammalian cochlea HCs from birth to adulthood, and that this expression was markedly decreased in both cochlear HCs and HEI‐OC1 cells following teicoplanin exposure, suggesting that TIGAR is involved in the teicoplanin injury process. Furthermore, overexpression of TIGAR restored cell viability, decreased apoptosis and reversed the reduction of Bcl-2/Bax ratio, whereas knockdown of TIGAR decreased cell viability, exacerbated apoptosis, and elevated Bcl-2/Bax ratio in HEI-OC1 cells following teicoplanin injury (Figs. 4 and 5). These results indicate that the overexpression of TIGAR alleviates teicoplanin injury, while TIGAR knockdown renders HEI-OC1 cells more susceptible to teicoplanin damage. This finding, together with our previous study that TIGAR overexpression protects spiral ganglion neurons against cisplatin injury [21], demonstrates that TIGAR may exert an oto-protective role in multiple inner ear cells against various types of damage.

Previous studies have shown that oxidative stress is a key mechanism underlying glycopeptide antibiotic induced cytotoxicity [36–38]. Here, we observed that teicoplanin caused ROS accumulation in HEI-OC1 cells, and the overexpression of TIGAR decreased the cellular level of ROS and the loss of ΔΨm in HEI-OC1 cells exposed to teicoplanin, whereas knockdown of TIGAR had opposing effect (Fig. 6). Rescue experiments showed that NAC alleviated the elevation of ROS and the depletion of ΔΨm following TIGAR knockdown, hence raising the cell viability and reducing apoptosis in HEI-OC1 cells damaged by teicoplanin (Fig. 7). These findings demonstrated that TIGAR modulates cellular redox status and alleviates the teicoplanin-induced ROS accumulation to protect HEI-OC1 cells against apoptosis. It has been demonstrated that TIGAR could significantly reduce intracellular ROS via its enzymatic effect, as it directs glucose to PPP and increases the amount of NADPH and 5-ribose phosphate, thereby produce reduced GSH or Trx (SH)2 and effectively eliminate ROS [12, 33]. Besides, TIGAR also regulates the mitochondrial function, cell survival and inflammation via regulating certain signal proteins through its non-enzymatic actions. For example, TIGAR is transported to mitochondria and interacts with HK2 [13], or ATP5A1 [39], through a mechanism independent of its FBPase activity, to reduce mitochondrial ROS and maintain cell survival.

MAPK family is essential for regulating cellular processes such as cell proliferation, differentiation, and apoptosis. P38 MAPK respond to a variety of extracellular stimuli including hypoxia, pro-inflammatory cytokines, and oxidative stress [29, 40, 41]. Studies reveal that P38 plays a crucial role in mediating ototoxicity caused by radiation, noise or aging [42–44]. In the present study, the phosphorylation of P38 protein was elevated in HEI-OC1 cells following teicoplanin damage, and it was further intensified when TIGAR was knocked down by shRNA, whereas it was alleviated by NAC (Fig. 7). Consequently, our findings suggest that the activation of P38 MAPK signaling pathway is implicated in teicoplanin-induced injury of HEI-OC1 cells and may represent a molecular mechanism underlying the effect of TIGAR on reducing oxidative stress and apoptosis generated by teicoplanin. Notably, although one study has reported that TIGAR deficiency promotes activation of ERK signaling, another major subfamily of MAPK, and thus supports the invasive capacity of pancreatic ductal adenocarcinoma cells [45], the relationship between TIGAR and P38 MAPK signaling pathway has not yet been established. Further research is required to investigate the role and the potential mechanisms underlying P38 MAPK signaling in teicoplanin ototoxicity, in particular the regulatory effect of TIGAR on P38 MAPK pathway, preferably using transgenic mice with altered expression of TIGAR or P38 in cochlea HCs.

In conclusion, the results of this study revealed the cytotoxicity of teicoplanin in both cochlea HCs and HEI-OC1 cells, and presented the protective effect of TIGAR on reducing HEI-OC1 cell apoptosis and oxidative stress following teicoplanin exposure. This protective mechanism may be associated with the regulation of P38 MAPK signaling pathway. Our findings imply that TIGAR could be a potential target for protecting HCs from teicoplanin-induced ototoxic damage.

## Supplementary Information

Below is the link to the electronic supplementary material.

Supplementary file1 (DOCX 1740 KB)

## Data Availability

The datasets used and/or analyzed during the current study are available from the corresponding author on reasonable request.
