# Supplementary material for: TIGAR Protects Cochlear Hair Cells against Teicoplanin-Induced Damage
Source: Mol Neurobiol. 2023 Mar 21;60(7):3788–802. doi: 10.1007/s12035-023-03309-8 (PMC10029784; doi:10.1007/s12035-023-03309-8)
Supplement: Supplementary file 1 — Supplementary file1 (DOCX 1740 KB) [file 12035_2023_3309_MOESM1_ESM.docx]

**
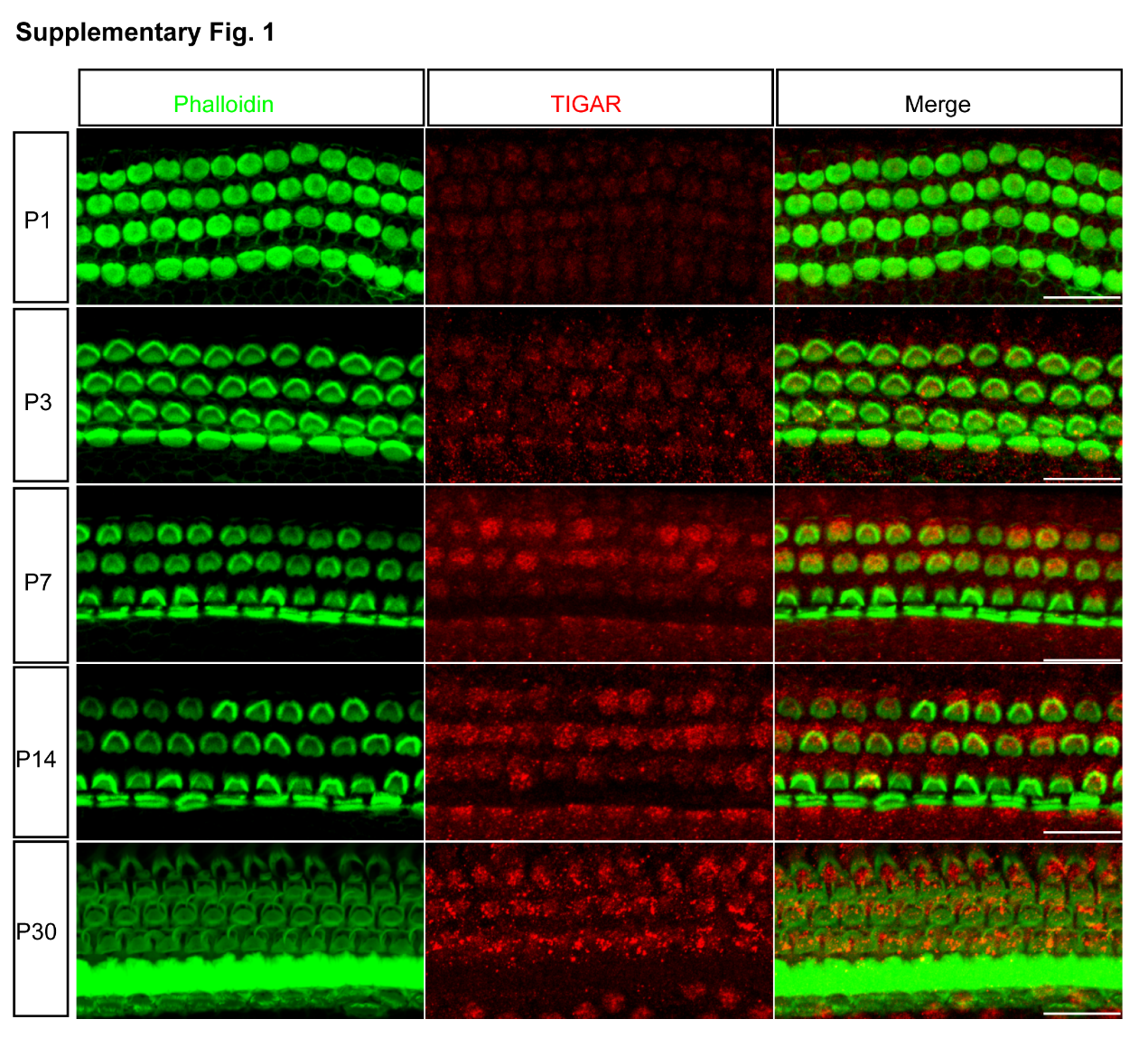
**

**Supplementary Fig. 1 The expression of TIGAR in postnatal cochlea HCs.** The expression of TIGAR in postnatal cochlea HCs was characterized with inner ears of C57BL/6 mice at different ages (P1, 3, 7, 14, 30). Representative immunostaining images showed that clear expression of TIGAR (red) was presented in middle turn cochlea HCs (phalloidin, green) from P1 to P30. Scale bar = 20 μm.


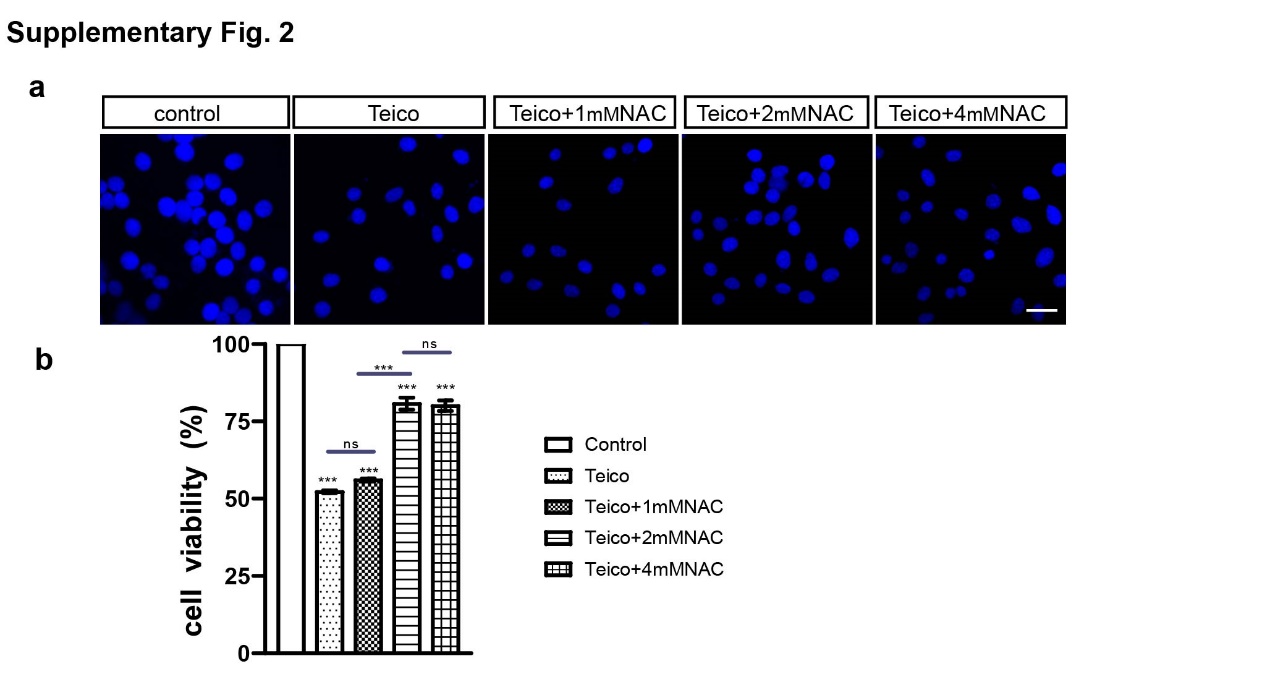


**Supplementary Fig. 2 The dose responses of NAC in teico-injured HEI-OC1 cells. a.** Representative immunostaining images of HEI-OC1 cells (DAPI, blue) with different concentrations (1, 2, 4 mM) of NAC pre-treatments for 2h, respectively, before 7.5mM teicoplanin treatment. **b**. The CCK-8 results showed that both 2 mM and 4 mM NAC pre-treatments successfully rescued the HEI-OC1 cell loss from teicoplanin damage, while 1mM NAC pre-treatment failed to increase the survived HEI-OC1 cell number compared to the teicoplanin-only group. The cell viability in 4 mM NAC pre-treatment group was not significantly different compared with that in the 2 mM NAC pre-treatment group, and thus the 2 mM NAC pre-treatment condition was chosen in the subsequent experiments. ** *P* < 0.01, *** *P* < 0.001. Scale bar = 20 μm.
